# A Novel Missense Variant of *BMPR1A* in Juvenile Polyposis Syndrome: Assessment of Structural and Functional Alternations

**DOI:** 10.1155/humu/7317429

**Published:** 2025-02-18

**Authors:** Mengyuan Yang, Ziyan Tong, Zhijun Yuan, Bingjing Jiang, Yingxin Zhao, Dong Xu, Ying Yuan

**Affiliations:** ^1^Department of Medical Oncology, Key Laboratory of Cancer Prevention and Intervention, Ministry of Education, The Second Affiliated Hospital, Zhejiang University School of Medicine, Hangzhou, Zhejiang, China; ^2^Cancer Institute, Key Laboratory of Cancer Prevention and Intervention, Ministry of Education, The Second Affiliated Hospital, Zhejiang University School of Medicine, Hangzhou, Zhejiang, China; ^3^Department of Radiation Oncology, Key Laboratory of Cancer Prevention and Intervention, Ministry of Education, The Second Affiliated Hospital, Zhejiang University School of Medicine, Hangzhou, Zhejiang, China; ^4^Department of Pathology, Affiliated Jinhua Hospital, Zhejiang University School of Medicine, Jinhua, Zhejiang, China; ^5^Department of Colorectal Surgery and Oncology, Key Laboratory of Cancer Prevention and Intervention, Ministry of Education, The Second Affiliated Hospital, Zhejiang University School of Medicine, Hangzhou, Zhejiang, China; ^6^Zhejiang Provincial Clinical Research Center for Cancer, Hangzhou, Zhejiang, China; ^7^Cancer Center of Zhejiang University, Hangzhou, Zhejiang, China; ^8^Binjiang Institute of Zhejiang University, Hangzhou, Zhejiang, China

**Keywords:** *BMPR1A*, juvenile polyposis syndrome, missense variant, pathogenic

## Abstract

Juvenile polyposis syndrome (JPS) is a rare precancerous condition associated with a high susceptibility to colorectal cancer. The genetic basis of JPS has been reported to lie in germline mutations in BMPR1A or SMAD4, resulting in diverse clinical manifestations and an elusive underlying mechanism. We firstly utilized a 139-gene next-generation sequencing (NGS) panel to detect the germline variants and further employed various prediction tools to assess the pathogenicity and functional alternations. Consequently, we identified a novel pathogenic BMPR1A missense variant (c.355C>T; p.R119C). More importantly, we proposed for the first time that the missense variant would lead to a decrease in molecular weight, potentially associated with reduced protein stability, diminished posttranslational modifications, and aberrant alternative splicing. These findings may provide novel perspectives for further exploration into the role of BMPR1A in JPS development. Also, we hope to encourage clinicians to underscore the importance of genetic testing and analysis in facilitating the diagnosis and treatment of diseases.

## 1. Introduction

Juvenile polyposis syndrome (JPS, OMIM Code 174900) is a rare autosomal dominant precancerous predisposition syndrome, with an incidence of approximately 1/100,000 [1, 2], typically resulting in multiple juvenile polyps in the colorectum (98%), stomach (14%), duodenum (7%), and small bowel (7%) [3]. These polyps significantly increase the risk of colorectal cancer, with a cumulative risk of up to 68% [4]. Germline disease-causing variants (DCVs) in the *SMAD* family member 4 (*SMAD4*) or bone morphogenetic protein receptor 1A (*BMPR1A*) genes have been reported in over 50% of JPS patients, with *BMPR1A* DCVs accounting for 17%–38% [5]. Despite the involvement of both proteins in the transforming growth factor-*β* (TGF-*β*)/bone morphogenetic protein (BMP) signaling pathway, these DCVs often result in distinct clinical presentations. JPS patients with *SMAD4* mutations predominantly exhibit upper gastrointestinal polyps and hereditary hemorrhagic telangiectasia (HHT), while *BMPR1A*-mutated patients mainly present with colonic phenotypes [6]. It follows that the highly variable clinical phenotypes of JPS can be partially explained by these DCVs, including polyp locations, extraintestinal manifestations, and risk of malignant transformation [7]. However, the exact mechanism by which these DCVs influence the development of JPS has not been extensively investigated [1, 8, 9].

The BMPR1A protein, a serine–threonine kinase type I receptor of the TGF-*β*/BMP superfamily, regulates colonic epithelial differentiation through activation of SMAD4-mediated intracellular signaling [10]. Mutations in *BMPR1A* are associated with a variety of disorders including JPS, idiopathic pulmonary arterial hypertension [11], primary ovarian insufficiency [12], and abnormal skeletal development [13]. Currently, studies on the association between *BMPR1A* and JPS primarily focus on identifying novel DCVs and establishing the gene–phenotype correlation. In JPS, DCVs of *BMPR1A* encompass missense/nonsense variants, small insertions/deletions, and large genomic deletions [14, 15]. These variants tend to disrupt the TGF-*β*/BMP pathway and impact cell proliferation, playing a crucial role in pathogenesis [16]. Also, patients with contiguous deletions of phosphatase and tensin homolog (*PTEN*) and *BMPR1A* tend to develop JPS at an earlier age with more severity, indicating a synergistic effect of contiguous deletions [17, 18]. However, limited research has been conducted on specific pathogenic mechanisms. In *Bmpr1a*-mutant mice, polyps exhibited cystically dilated glands filled with mucin (a JPS-like phenotype), demonstrating the potential function of *BMPR1A* in a mouse model [19]. Furthermore, a functional study has suggested that certain *BMPR1A* missense mutations identified in JPS result in alterations to protein conformation and affect cellular localization [20]. Additionally, a novel nonsense variant (c.1114A>T, p.Lys372Ter) was found to cause nonsense-mediated mRNA decay (NMD), thus inhibiting BMP signaling activity [21]. More comprehensive functional experiments are necessary to validate the role of the *BMPR1A*-related signaling pathway in polyp formation and carcinogenesis of JPS.

Here, we present a case of JPS with *BMPR1A* missense variant (c.355C>T; p.R119C), proposing for the first time that this variant may lead to a decrease in molecular weight and can be interpreted as pathogenic.

## 2. Materials and Methods

### 2.1. Patients and DNA Samples

The study was approved by the Ethics Committee of the Second Affiliated Hospital of Zhejiang University School of Medicine (Approval No. 2017.066). Written informed consent was obtained from this patient.

Genomic DNA was extracted from peripheral EDTA-anticoagulated blood samples using the standard procedure (QIAamp DNA blood midi kit, Qiagen, Hilden, Germany).

### 2.2. Next-Generation Sequencing (NGS) and Analysis

The NGS panel covers the whole exon region of 139 genes associated with 16 cancers including colorectal cancer and 70 genetic syndromes, which was performed by Genetron Health (Beijing, China) on the HiSeqX-ten sequencing platform (Illumina, San Diego, United States). These 139 genes have been described in previous study [22]. We classified germline variants according to the American College of Medical Genetics and Genomics (ACMG) standards and guidelines for sequence variant interpretation [23].

### 2.3. Functional Experiments

#### 2.3.1. Cell Culture

L293T cells were purchased from ATCC and were cultured at 37°C in 5% CO_2_ in the RPMI medium 1640 supplemented with 10% fetal bovine serum (FBS) (Gibco).

#### 2.3.2. Construction of Overexpression Plasmid

The human *BMPR1A* (NM_004329) cDNA cloning was purchased from YouBio (Hunan, China), which was connected to the pcDNA3.1-3xflag according to the manufacturer's instructions. The missense variant c.355C>T was mutated through PCR amplification. The human *BMP2* (NM_001200) cDNA cloning was purchased from YouBio (Hunan, China), which was tagged with 6xHis and then was connected to pcDNA3.1 according to the manufacturer's instructions.

#### 2.3.3. Coimmunoprecipitation (CoIP)

The pcDNA3.1-BMP2-6xHis and pcDNA3.1-BMPR1A wildtype-3xflag or pcDNA3.1-BMPR1A mutant-3xflag plasmids were transfected into L293T cells at a 1:1 ratio. Transient transfection was performed by LipoD293 In Vitro DNA Transfection Reagent (SignaGen Laboratory, Rockville, Maryland, United States). Forty-eight hours after transfection, whole-cell extracts were lysed in IP buffer with protease inhibitor and incubated on ice for 30 min. After centrifugation for 15 min at 14,000 *g*, supernatants were collected and incubated with ANTI-FLAG M2 magnetic beads (M8823, Sigma-Aldrich, Darmstadt, Germany). After incubation overnight, beads were washed three times with immunoprecipitation buffer. After discarding the supernatant, add 1× loading buffer, and heat at 100°C for 5 min; the supernatant was magnetically separated for subsequent WB assay and blotted with mouse anti-FLAG (M2, F3165, Sigma-Aldrich, 1:1000) and mouse anti-6xHis (HIS.H8, Invitrogen, 1:1000).

#### 2.3.4. Glycosylation Detection

The *BMPR1A* wildtype and mutant plasmids were transfected into L293T cells, respectively. Transient transfection was performed by LipoD293 In Vitro DNA Transfection Reagent (SignaGen Laboratory, Rockville, Maryland, United States). Forty-eight hours after transfection, cell proteins were extracted in 1× denaturing buffer. Prior to immunoblotting, samples were treated with N-glycosidase (N-Gly) F (PNGase F, P0704S) to remove N-glycans or O-glycosidase (O-Gly) (P0733S) and *α*2-3,6,8 neuraminidase (P0720S) to remove O-glycans, and these enzymes are all purchased from New England Biolabs (Beverly, Massachusetts). And then, the samples were blotted with mouse anti-FLAG (M2, F3165, Sigma-Aldrich, 1:1000) and rabbit anti-Vinculin (ET1705-94, Huabio, 1:1000).

### 2.4. Assessment of Functional and Structural Consequences

#### 2.4.1. Prediction of Pathogenicity and Molecular Mechanisms

We utilized 13 prediction tools to evaluate the pathogenicity of *BMPR1A* p.R119C variant, including SIFT, PolyPhen-2, PROVEAN, P-Mut, Mutation_Assessor, CADD, REVEL, VEST4, FATHMM, GERP++, BayesDel, MPC, and PrimateAI. The SIFT algorithm evaluates how alterations in amino acid residues affect protein function and phenotype as an indicator of pathogenicity [24]. PolyPhen-2 allows physical comparison and potential categorization based on various sequence and structural features at substitution positions [25]. PROVEAN sequentially compares mutated protein to a reference sequence [26]. P-Mut could distinguish between neutral and disease-associated protein sequences [27]. Mutation_Assessor is based on evolutionary conservation of the affected amino acid in protein homologs [28]. CADD is a framework that combines various annotations to generate a single quantitative score [29]. REVEL is an ensemble method used to predict the pathogenicity of missense variants by utilizing individual tools [30]. VEST4 is a machine learning–based classifier designed to prioritize rare missense variants that may be associated with human disease [31]. FATHMM is a species-independent method that can predict the functional effects of protein missense variants, with the option of incorporating species-specific weightings [32]. GERP++ is a tool that utilizes maximum likelihood evolutionary rate estimation to provide position-specific scoring [33]. BayesDel is a versatile tool used for gene prioritization in gene discovery research and variant classification in clinical genetic testing [34]. PrimateAI aids in systematically identifying pathogenic mutations through a process of elimination [35]. MutPred2 is an independent sequence homology–based machine learning software that examines the structural, functional, and phenotypic outcomes of variants to forecast potential disease-causing molecular mechanisms [36]. Our predicted scores and pathogenicity results are based on the 2022 ClinGen calibration study [37].

#### 2.4.2. Prediction of Structure and Binding Affinity

The protein structure of BMPR1A wild-type (WT) and the R119C mutant, including hydrogen bonds and disulfide bonds, was predicted using AlphaFold3 [38]. For each input, the model generated five predictions, and the predictions with the highest overall structure confidences were used for further analysis. All analyzed structures exhibited a predicted template modeling (pTM) score greater than 0.5, indicating that the overall predicted fold for the complex might be similar to the true structure [39].

The interaction structure of BMPR1A protein and BMP2 was predicted by AlphaFold3. Then, the predictions were visualized in ChimeraX for structural analysis including hydrogen bonds and contacts [40]. Additionally, PRODIGY, a software designed to predict binding affinity in biological complexes, was employed to further forecast protein–protein interactions [41].

#### 2.4.3. Prediction of Protein Modifications and Alternative Splicing

Protein stability prediction was conducted using I-Mutant 2.0 [42], MUpro [43], FoldX [44], Rosetta [45], GeoDDG [46], DynaMut2 [47], DDMut [48], INPS-MD [49], DUET [50], mCSM [51], and SDM [52]. For FoldX, Rosetta, and GeoDDG, it is emphasized that negative DDG values indicate increased stability [53]; however, apart from them, all other prediction tools directly suggest that DDG < 0 indicates decreased stability [54].

For O-glycosylation prediction, we employed the NetOglyc 4.0 server to predict mucin-type O-glycosylation sites in mammalian proteins [55]; a score > 0.5 suggests positive effects. We also utilized updated tools including ISOGlyP and HOTGpred. ISOGlyP calculates the enhancement value product (EVP) as an indicator of glycosylation rate; an EVP value > 1 indicates a higher likelihood of glycosylation [56]. HOTGpred is a machine learning model for prediction, assigning a value to the probability of glycosylation for each protein [57]. For N-glycosylation prediction, we utilized the NetNglyc 1.0 server to identify potential N-glycosylation sites through artificial neural networks [58].

For ubiquitination and phosphorylation prediction, GPS-SUMO was applied to forecast sumoylation sites and SUMO interaction motifs in proteins [59]. Kinase prediction was conducted using NetPhos 3.1, a tool that employs an ensemble of neural networks to predict serine, threonine, or tyrosine phosphorylation sites in proteins [60].

Additionally, MusiteDeep is an online resource providing a deep learning framework for protein posttranslational modification (PTM) site prediction and visualization, including glycosylation, ubiquitination, and phosphorylation [61]. Sitetack is also an updated deep learning model that improves PTM prediction by using known PTMs [62].

For alternative splicing predictions, we employed the Human Splicing Finder (HSF) as a tool for predicting the impact of mutations on splicing signals or identifying splicing motifs in human sequence [63]. SpliceAI [64, 65] was also employed to predict splicing alterations. Its delta scores, ranging from 0 to 1, represent the probability that the variant influences splicing at any position.

## 3. Results

### 3.1. A Novel Pathogenic Missense Variant of *BMPR1A* in JPS

**Figure 1:**
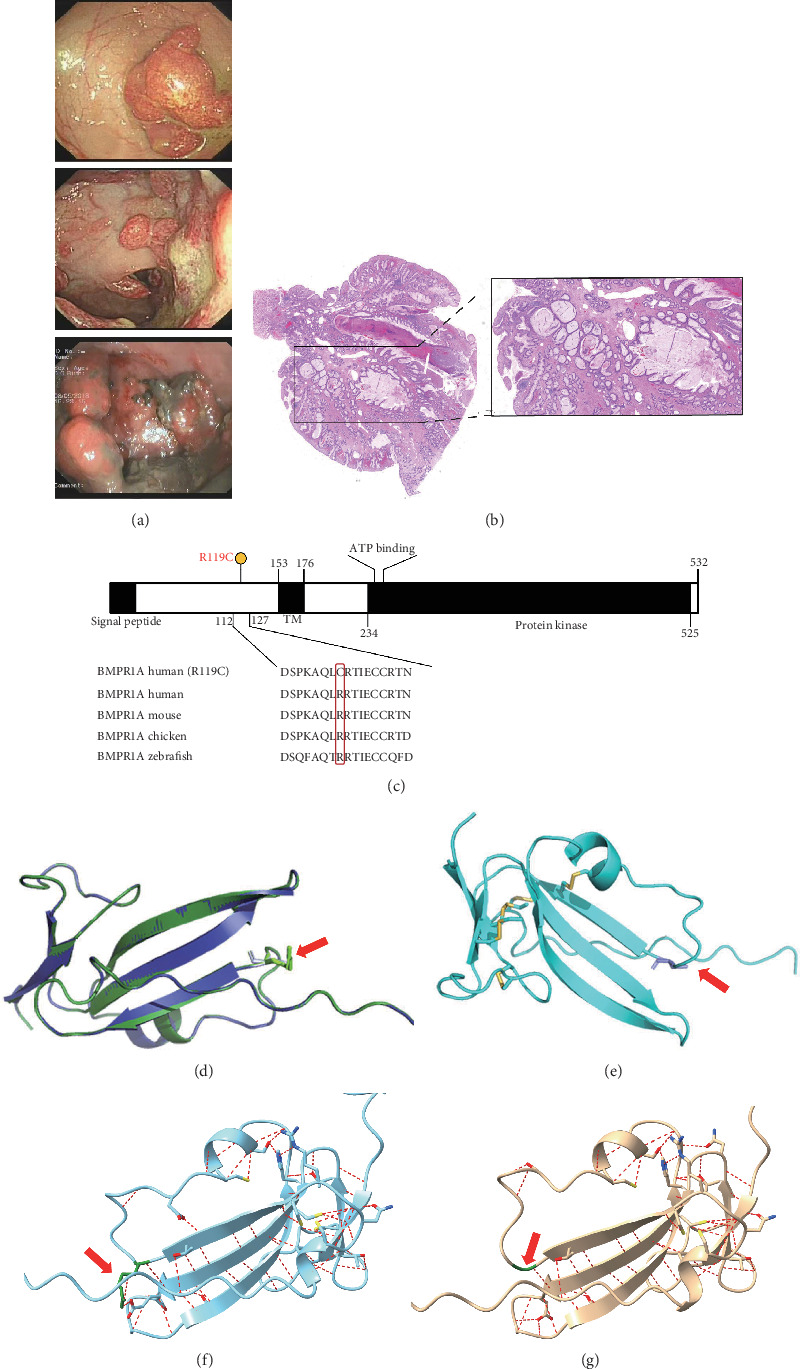
Clinicopathological and genetic information of the patient. (a) Endoscopy findings at the initial diagnosis. (b) HE staining of the colorectal polyps. (c) Schematic BMPR1A structure and the missense variant (p.R119C) is depicted in a lollipop plot with a high degree of conservation among different species. (d) Three-dimensional models of the wild-type (blue) and mutant (green) domains. For both wild-type and mutant predicted structures, pTM = 0.63. (e) Distribution of disulfide bonds (yellow) around the mutation site (red arrow). Distribution of hydrogen bonds (red dashed line) around the mutation site (red arrow) of wild-type BMPR1A protein (f) and p.R119C (g).

He was a 15-year-old male patient without any family history of cancer or polyposis, and his first colonoscopy showed that the entire colorectum is scattered with multiple polyps and there are more than 100 polyps in total; the largest is about 5∗5 cm in the rectum (Figure 1(a)). The patient has undergone multiple endoscopic polypectomy procedures, and most of the polyps were diagnosed pathologically as juvenile polyps (Figure 1(b)).

NGS showed that the patient carried a missense mutation in *BMPR1A* gene (c.355C>T; p.R119C), which is not included in multiple population databases (1000_CN, 1000_MAF, ESP6500, ExAC), and some literatures reported that this variant had been detected in families related to JPS [14]. This missense variant is located in the extracellular segment of the protein encoded by *BMPR1A* and is highly conserved in multiple species (Figure 1(c)). However, we performed 3D modeling analysis of the WT and mutant extracellular domains and found that the change of amino acid at Position 119 did not significantly affect the overall protein structures (Figure 1(d)) and the distribution of surrounding disulfide bonds (Figure 1(e)), albeit with a slight adjustment in hydrogen bonds (Figures 1(f) and 1(g)).

Next, in order to assess the pathogenicity of this missense variant, we employed various bioinformatics prediction tools to anticipate its potential functional implications. Positive results were obtained from 11 out of 13 widely used prediction tools (Table 1), indicating a high likelihood that the missense variant (p.R119C) might induce pathogenic alterations. Additionally, we utilized MutPred2, a predictive software for forecasting disease-causing molecular mechanisms triggered by SNP-induced amino acid substitutions. The results showed that *BMPR1A* p.R119C may be related to altered metal binding and altered transmembrane protein; furthermore, it may lead to gain of disulfide linkage at C124 and pyrrolidone carboxylic acid at Q117 (Table 1). These results present confident hypotheses for the possible pathogenicity as well as functional changes that may result from the mutation.

### 3.2. Prediction and Validation of the Interaction Between BMPR1A and BMP2

**Figure 2:**
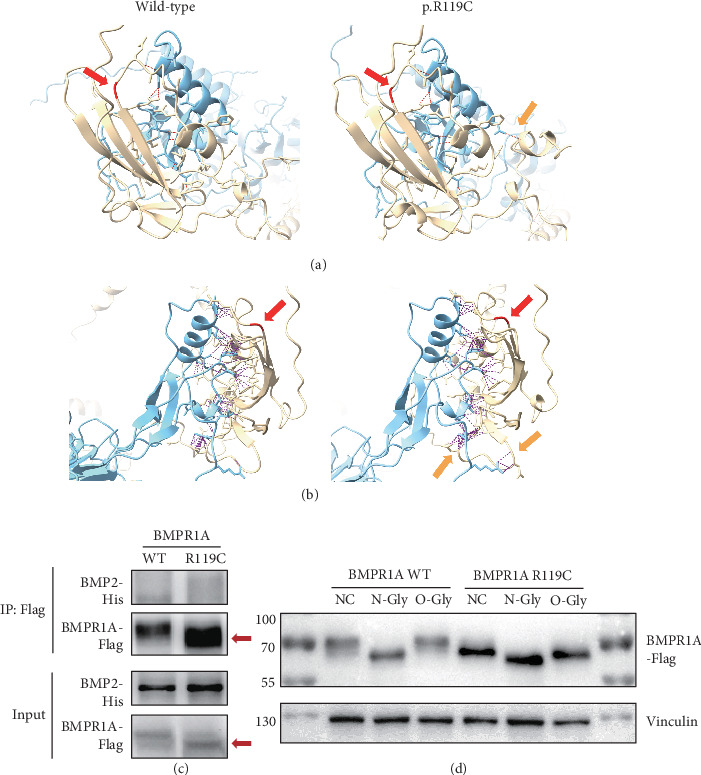
A lower molecular weight of the missense variant (p.R119C) than the wild-type BMPR1A protein. (a) Structural prediction of BMPR1A-BMP2 interaction: hydrogen bonds (red dashed line) change (orange arrows) around the mutation site (red arrow). (b) Structural prediction of BMPR1A-BMP2 interaction: contacts (purple dashed line) change (orange arrows) around the mutation site (red arrow). (c) Coimmunoprecipitation (CoIP) to evaluate the interaction between BMPR1A and BMP2 protein. (d) Immunoblotting of wild-type (WT) and mutated (R119C) BMPR1A with nontreated (NC), N-glycosidase-treated (N-Gly), and O-glycosidase (O-Gly).

Previous studies have indicated that BMP2 interacts with BMPR1A and triggers the downstream TGF-*β*/BMP pathway [66]. Therefore, we utilized AlphaFold3 to model the structure of the BMPR1A-BMP2 interaction in order to predict whether *BMPR1A* p.R119C affects the interaction force between the proteins. The results showed that the mutation resulted in an increased protein binding affinity of BMPR1A with BMP2 (Table 2) and more interprotein hydrogen bonds and contacts (Figures 2(a) and 2(b)). It suggested that this missense variant might lead to aberrant overactivation of the TGF-*β*/BMP pathway. We then performed CoIP to verify protein interactions. Unfortunately, we did not observe satisfactory results for BMP2 binding. However, we thus discovered an interesting phenomenon that the variant (p.R119C) exhibited a lower molecular weight than the WT BMPR1A protein (Figure 2(c)). It has never been reported before, and we believe that it may also be a major cause of altered function. We then conducted further predictions and experimental analyses.

### 3.3. A Lower Molecular Weight in the BMPR1A p.R119C Variant

Changes in protein molecular weight are highly probably associated with PTMs, particularly glycosylation modifications. Therefore, we first carried out experiments to explore whether *BMPR1A* p.R119C was associated with altered glycosylation modifications. Results indicated that, after treatment with N-Gly or O-Gly, the missense variant may affect the N-glycosylation of the protein and have a modest effect on O-glycosylation (Figure 2(d)). Subsequently, we also performed the prediction of protein stability (Table 3) and various types of protein modifications (Table 4). The prediction results of protein stability indicated that 8 out of 11 prediction tools, including Rosetta, predicted a decrease in the stability of the mutated BMPR1A protein. In contrast, FoldX and GeoDDG showed a slight increase in stability. These findings suggest that the overall stability of the mutated protein may exhibit a slight decrease; however, there is still potential bias among different prediction results. Regrettably, no significantly plausible glycosylation alterations were identified across different prediction tools, apart from a minor change at one O-glycosylation site as observed within our experimental findings. However, through two updated deep learning frameworks for PTM prediction, namely, Sitetack and MusiteDeep, we found that its decreased phosphorylation level may also contribute to the decreased molecular weight of the protein.

Abnormalities in variable splicing may also bring about changes in molecular weight. Therefore, we utilized HSF and SpliceAI to further predict the potential splicing alterations induced by p.R119C (Table 5). The findings indicated that the mutation does not reside at the crucial splice site, thereby not significantly affecting the acceptor and donor sites; however, it may introduce a new exon splicing enhancer (ESE) site and disrupt two original exon splicing silencer (ESS) sites (Table 5). This implies that the mutation could also result in splicing disruption and potentially cause an early stop codon, thereby leading to a decrease in protein molecular weight. Thus, *BMPR1A* p.R119C may affect protein function through influencing variable splicing, as well as glycosylation and phosphorylation modifications.

## 4. Discussion

The *BMPR1A* p.R119C variant has been previously documented [14, 67]. A recent publication also proposed this missense variant in JPS with NGS and further demonstrated its potential pathogenicity through prediction tools like PolyPhen-2 [67]. However, they failed to offer a more in-depth analysis of the pathogenic mechanisms. In our case, this missense variant can be defined as pathogenic, according to the ACMG standard (Table 6).

The BMPR1A receptor is a crucial component protein of the TGF-*β*/BMP pathway, working in conjunction with other receptors such as BMPR1B and BMPR2, to synergistically receive signals from extracellular growth factors like BMP2, BMP4, and BMP6. This leads to pathway activation, with phosphorylation of SMAD1/5/8 molecules followed by complex formation with SMAD4 to regulate transcription of downstream target genes [68]. The BMP signaling pathway plays a vital role in stabilizing intestinal homeostasis and normal differentiation of the intestinal epithelium [69]. Abnormal disruption of the signaling pathway can largely impact normal proliferation of intestinal cells, as well as the tumor microenvironment balance [70]. Studies have shown that JPS development is closely associated with the BMP pathway. Sarah et al. have demonstrated that loss of BMPR1A-mediated signaling in fibroblasts could lead to the formation of serrated intestinal polyps [71]. However, its further specific pathogenic mechanisms remain unclear, including the precise impact of inactivation or overactivation of signaling pathways on the pathogenesis of JPS. In our CoIP results, we observed that the *BMPR1A* p.R119C variant did not affect BMP2 binding. Instead, we noted a decrease in the molecular weight. This implicated that the missense variant might disrupt the BMP pathway through other potential molecular mechanisms, ultimately resulting in JPS. This prompted us to conduct further investigation.

We are the first to propose that this *BMPR1A* missense variant may result in a decrease in protein molecular weight. As a prevalent PTM, glycosylation plays a crucial role in various biological processes such as protein stability, signal transduction, and cell–cell interactions [72, 73]. The formation of dysfunctional glycans is associated with disease states [74, 75]. Also, changes in glycosylation modifications can impact the three-dimensional structure and interaction affinity between the receptor and ligand [76]. Jonathan et al. have demonstrated that N-glycosylation on the BMPR2 receptor may specifically enhance BMP2 binding, which could mediate developmental and tissue homeostasis [77]. This provides substantial evidence for the potential impact of glycosylation modifications on protein function. As indicated in our findings, the variant might result in a reduction of protein N-glycosylation and slightly affect O-glycosylation. Our predictive results suggested that the variant might lead to a slight decreased protein stability, which aligns with the well-established role of glycosylation in maintaining protein conformation [78]. Although we did not observe a significant difference in subsequent glycosylation predictions, we still have grounds to argue that *BMPR1A* p.R119C is likely associated with an alteration in glycosylation modification, potentially leading to changes in protein conformation and molecular weight, ultimately impacting protein function.

Phosphorylation frequently induces conformational changes in proteins and plays a crucial regulatory role in various intracellular processes, including growth, proliferation, and cell division. Recent studies have elucidated the mechanisms by which aberrant phosphorylation contributes to cancer development [79–81]. Specifically, the gain or loss of phosphorylation sites has been identified as a crucial mechanism through which dysregulated phosphorylation impairs signal transduction [82]. Our results indicate that this mutated protein may exhibit reduced phosphorylation levels, which could influence its molecular weight. This finding further suggests that the R119C variant could lead to abnormalities in PTM processes, thereby affecting the normal function of this protein receptor and disrupting signal transduction.

Furthermore, we observed certain changes in mRNA splicing. Abnormal splicing may result in alternations within crucial functional protein domains, thus leading to their impaired functionality. Presently, numerous cancer-specific alternative splicing forms have been identified as pivotal factors influencing tumor progression and invasion [83], including colorectal malignancies [84]. A recent finding indicated that a newly identified *BMPR1A* mutation (c.778+5G>C) in JPS could influence mRNA splicing [85], suggesting that irregularities in alternative splicing might significantly contribute to the formation of intestinal polyps in JPS. However, they did not delve further into the specific effects of this variant on BMPR1A protein function. This prompted us to predict the splicing pattern for the R119C variant. While this particular locus does not affect the crucial splice site directly, we have a basis for arguing that it might disrupt regulators of alternative splicing based on the results of HSF. If the abnormal regulation results in frameshift and premature termination codons, then the observed reduction in protein molecular weight can be partially accounted for.

Overall, we have effectively demonstrated the pathogenicity of the *BMPR1A* R119C variant and comprehensively predicted its structural and functional alternations. Although our predictions and experimental investigations are still limited, the phenomenon of reduced protein molecular weight, initially proposed by us, could offer new insights for subsequent researchers to explore the pathogenic mechanism of BMPR1A protein in JPS.

## 5. Conclusion

In summary, we demonstrated that *BMPR1A* p.R119C is highly likely to be pathogenic. More importantly, we proposed for the first time that the missense variant would lead to a decrease in molecular weight, potentially associated with reduced protein stability, diminished PTMs, and aberrant alternative splicing. These findings may provide novel perspectives for further exploration into the role of *BMPR1A* in JPS development.

## Figures and Tables

**Table 1:** Prediction of pathogenicity and functional consequences of *BMPR1A* R119C.

**Predictive tool**	**Score**	**Threshold**	**Result**
SIFT	0.091	< 0.05	Tolerated
PolyPhen-2	0.988	> 0.85	Probably damaging
PROVEN	−2.04	< −2.5	Neutral
P-Mut	0.68	> 0.5	Disease
Mutation_Assessor	0.81802	/	Functional (median)
CADD	28.9	> 25.3	Pathogenic
REVEL	0.825	> 0.644	Pathogenic
VEST4	0.924	> 0.764	Pathogenic
FATHMM	−4.50	< −4.14	Damaging
GERP++	4.24	> 2.70	Pathogenic
BayesDel	0.4658	> 0.13	Pathogenic
MPC	1.407	> 1.360	Pathogenic
PrimateAI	0.793	> 0.790	Pathogenic
	**Score**	**Molecular mechanisms**	**p** ** value**
MutPred2^#^	0.868	Altered metal binding	*p* = 0.02
		Altered transmembrane protein	*p* = 4.6e − 04
		Gain of disulfide linkage at C124	*p* = 1.2e − 03
		Gain of pyrrolidone carboxylic acid at Q117	*p* = 0.04

^#^MutPred2: score > 0.75 and *p* value < 0.05 are referred to as confident hypotheses.

**Table 2:** Prediction of binding affinity between BMPR1A and BMP2.

	**Predictive tool**	***BMPR1A* WT**	***BMPR1A* R119C**
Number of H-bonds	ChimeraX	15	16
Number of contacts		141	178
Binding affinity (Δ*G*)^∗^	PRODIGY	−14.3	−15.4
Dissociation constant (*K*_d_)^#^		8.3e − 11	1.4e − 11
ICs charged–charged		21	25
ICs polar–apolar		21	23

Abbreviations: Δ*G*, Gibbs free energy; ICs, number of intermolecular contacts; *K*_d_, dissociation constant.

⁣^∗^Δ*G* = *RT*ln*K*_d_, the smaller the Δ*G*, the more stable the complex is.

^#^ *K*_d_ < e − 08, strong binding; e − 08 < *K*_d_ < e − 04, moderate binding; *K*_d_ > e − 04, weak binding.

**Table 3:** Prediction of protein stability changes in *BMPR1A* R119C.

**Predictive tool**	**DDG (kcal/mol)**	**Result**
I-Mutant 2.0	−0.33	Decreased stability
MUpro	−0.72707561	Decreased stability
FoldX 5.1⁣^∗^	−2.81	Increased stability
Rosetta⁣^∗^	4.078	Decreased stability
GeoDDG⁣^∗^	−0.6338395476341248	Increased stability
DynaMut2	−0.31	Destabilizing
DDMut	−0.04	Destabilizing
INPS-MD	−0.32	Neutral
DUET	−0.577	Destabilizing
mCSM	−0.66	Destabilizing
SDM	−0.09	Destabilizing

*Note: *DDG = dG^Mutant^ − dG^WT^.

Abbreviations: DDG, free energy change.

⁣^∗^For FoldX, Rosetta, and GeoDDG, DDG < 0 indicates increased stability. In contrast, all other prediction tools suggest that DDG < 0 indicates decreased stability.

**Table 4:** Prediction of protein modification alterations in *BMPR1A* R119C.

	**Predictive tool**	**Score/probability**	**Result**
O-linked glycosylation	NetOglyc 4.0^&^	0.51 to 0.44 at Position 49	Decrease glycosylation
	ISOGlyP	/	Negative
	HOTGpred	/	Negative
	MusiteDeep⁣^∗^	/	Negative
	Sitetack⁣^∗^	/	Negative

N-linked glycosylation	NetNglyc 1.0^&^	/	Negative
	MusiteDeep⁣^∗^	/	Negative
	Sitetack⁣^∗^	/	Negative

Ubiquitination	GPS-SUMO	/	Negative
	MusiteDeep⁣^∗^	/	Negative
	Sitetack⁣^∗^	0.5668 to 0.541 at Position 115	Decrease ubiquitination

Phosphorylation	NetPhos 3.1 Server	/	Negative
	MusiteDeep⁣^∗^	0.785 to 0.714 at Position 113	Decrease phosphoserine
	Sitetack⁣^∗^	0.6438 to 0.5375 at Position 113	Decrease phosphoserine

⁣^∗^For MusiteDeep and Sitetack, the positive result is defined by a cutoff value of 0.5.

^&^For NetOglyc 4.0 and NetNglyc 1.0, a score > 0.5 suggests positive effects.

**Table 5:** Prediction of alternative splicing changes in *BMPR1A* R119C.

**Predictive tool**	**Score/probability**	**Result**
Human Splicing Finder	No new or broken acceptor/donor sites	Inhibition of splicing
	1 new ESS site	
	2 ESE sites broken	

SpliceAI⁣^∗^	Acceptor loss score = 0.01	Negative alternations
	Donor loss score = 0.02	
	No acceptor/donor gain	

Abbreviations: ESE, exon splicing enhancer; ESS, exon splicing silencer.

⁣^∗^For SpliceAI, details of the delta score cutoffs are as follows: 0.2 (high recall), 0.5 (recommended), and 0.8 (high precision).

**Table 6:** Classification of *BMPR1A* R119C missense variant according to the ACMG standard.

**Evidence**	***BMPR1A* R119C**	**Result**
Very strong, PVS1	Not applicable	Pathogenic (2PS +2 PM + 2PP)
Strong, PS1~4	Same amino acid alterations as previously reported pathogenic variant (PS1)	
	Impaired gene function with a decreased protein molecular weight (PS3)	
Moderate, PM1~6	Not included in the population database (PM2)	
	A novel variant without parental verification (PM6)	
Supporting, PP1~5	Predicted to be deleterious by various tools (PP3)	
	Reported as likely pathological before (PP6)	

## Data Availability

The data that support the findings of this study are available on request from the corresponding author. The data are not publicly available due to privacy or ethical restrictions.
